# Lipidomics profiling and circulating triglyceride concentrations in sub-Saharan African individuals

**DOI:** 10.1038/s41598-024-71734-3

**Published:** 2024-09-06

**Authors:** Amy R. Bentley, Ayo P. Doumatey, Jie Zhou, Lin Lei, Karlijn A. C. Meeks, Elisabeth F. Heuston, Charles N. Rotimi, Adebowale A. Adeyemo

**Affiliations:** Center for Research on Genomics and Global Health, National Human Genome Research Institute, National Institutes of Health, 12 South Drive, Building 12A Room 1025, Bethesda, MD 20892-5635 USA

**Keywords:** Lipidomics, Metabolomics, Dyslipidaemias

## Abstract

Elevated triglycerides (TG) are a risk factor for cardiometabolic disorders. There are limited data on lipidomics profiles associated with serum triglycerides concentrations, although these could advance our understanding of the mechanisms underlying these associations. We conducted a lipidomics study of 308 Nigerians with replication in 199 Kenyans. Regression models were used to assess the association of TG with 480 lipid metabolites. Association and mediation analyses were conducted to determine the relationship among TG, metabolites, and several cardiometabolic traits. Ninety-nine metabolites were significantly associated with TG, and 91% of these associations replicated. Overrepresentation analysis identified enrichment of diacylglycerols, monoacylglycerols, diacylglycerophosphoethanolamines, monoacylglycerophosphocholines, ceramide phosphocholines, and diacylglycerophosphocholines. TG-cardiometabolic trait associations were largely mediated by TG-associated metabolites. Associations with type 2 diabetes, waist circumference, body mass index, total cholesterol, and low-density lipoprotein cholesterol concentration were independently mediated by metabolites in multiple subpathways. This lipidomics study in sub-Saharan Africans demonstrated that TG is associated with several non-TG lipids classes, including phosphatidylethanolamines, phosphatidylcholines, lysophospholipids, and plasmalogens, some of which may mediate the effect of TG as a risk factor for cardiometabolic disorders. The study identifies metabolites that are more proximal to cardiometabolic traits, which may be useful for understanding the underlying biology as well as differences in TG-trait associations across ancestries.

## Introduction

Dyslipidemia is a hallmark of cardiometabolic dysfunction and, for this reason, concentrations of serum lipids, such as triglycerides (TG) are routinely assayed as clinical measures of cardiometabolic disease risk^1^. Alterations in serum lipids often precede the development of diabetes^2,3^ and atherosclerosis^4^. For instance, longitudinal analyses in American Indians demonstrated that changes in the plasma lipidome explained up to 3.0%, 4.0%, and 8.4% of the variability in fasting glucose, insulin resistance, and insulin sensitivity, respectively, over 5 years of follow-up^3^. Thus, deeper understanding of the mechanisms underlying elevated TG concentration is likely to give us greater insight into early stages of cardiometabolic dysfunction.

The interpretation of the strong associations of TG with cardiometabolic outcomes can be challenging. TG concentration is also correlated with many cardiometabolic biomarkers and risk factors, including high-density lipoprotein cholesterol concentration (HDL-c), low-density lipoprotein cholesterol concentration (LDL-c), fasting plasma glucose, and lifestyle. Thus, it can be difficult to discern whether observed associations with cardiometabolic disorders reflect an underlying causal role for TG or a relationship between TG and these other related quantities. Studies looking at genetic variants that are strongly associated with TG have been helpful in this regard, as the association between genetically influenced TG concentration is independent of confounding factors. Such studies suggest an independent, causal role of TG levels on cardiovascular disease and related traits^5–9^. A causal association of TG on glycemic traits (and of glycemic traits on TG) has also been observed^10,11^, but the overall evidence for T2D and related biomarkers is not consistent^11^, with a causal protective association of TG with T2D even being reported^12^.

An important challenge in interpreting the role of TG in cardiometabolic risk is that measured TG concentrations are a summation of a number of heterogenous molecules with properties varying depending on the chain lengths and saturation of the fatty acids within the TG molecule. Also, TG concentration represents the influence of variety of lipids pathways. A metabolomics approach, i.e. the comprehensive analysis of low molecular weight organic compounds, could help to identify the specific lipid metabolites that are associated with TG. In turn, this could help identify the pathways that are being perturbed as TG concentration is elevated, with the concomitant increase in cardiometabolic risk.

Research on the metabolites associated with increasing TG in African ancestry populations are of particular interest. Individuals of West African ancestry display generally lower TG concentrations compared to other populations, despite widely varying environmental backgrounds and without consistent differences in other serum lipid measurements (such as HDL-c or LDL-c)^13–15^. Also, associations between TG and some cardiometabolic risk factors, such as hypertension, body mass index (BMI), and waist circumference were weaker among individuals of West African vs. European ancestry^15^. A more fine-scaled look at lipid metabolites associated with TG may help us to understand the source of such heterogeneity and potentially identify metabolites that are more proximal to the cardiometabolic outcomes.

In this study, we utilized a lipidomics approach in the Africa America Diabetes Mellitus (AADM) study to evaluate the association of 480 lipid metabolites with TG concentration in 308 Nigerians, with replication in 199 Kenyans. We identified significant TG-associated lipid metabolites as well as the lipids sub-classes and pathways they represent. We evaluated the association between the most significant TG-associated lipid metabolites and several cardiometabolic traits. Finally, we conducted mediation analysis to evaluate the pattern and extent of mediation of the associations between TG and TG-associated cardiometabolic traits by the most significant TG-associated lipid metabolites.

## Results

We evaluated the association of lipid metabolites with TG in 308 Nigerian adults that included 77% women and 23% men (Supplementary Table S1).The subset of individuals without T2D (n = 208) had similar values for BMI (mean 32.2 [women]; 25.0 [men] kg/m^2^), TG (median 88.0 [women]; 81.0 [men] mg/dl), HDL-c (median 51.0 [women]; 44.4 [men] mg/dl), and LDL-c (median 129.5 [women]; 97.0 [men] mg/dl).

### Lipid metabolites association with TG

**Fig. 1:**
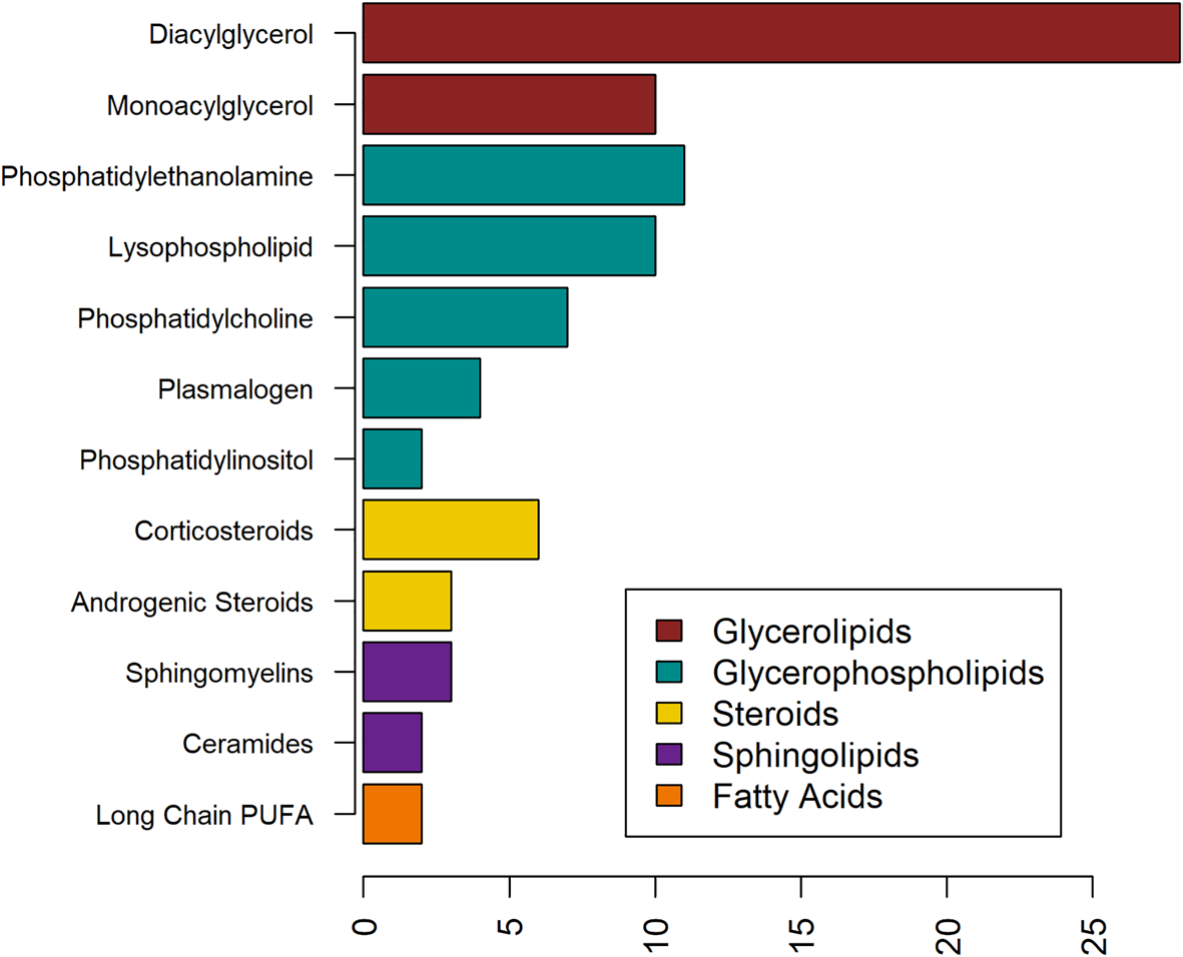
Subpathways of Metabolites Significantly Associated with TG. Distribution of associated metabolites by subpathway in analysis of TG. Only subpathways with n > 1 metabolites are shown. Full results are in Supplementary Table 2.

**Fig. 2:**
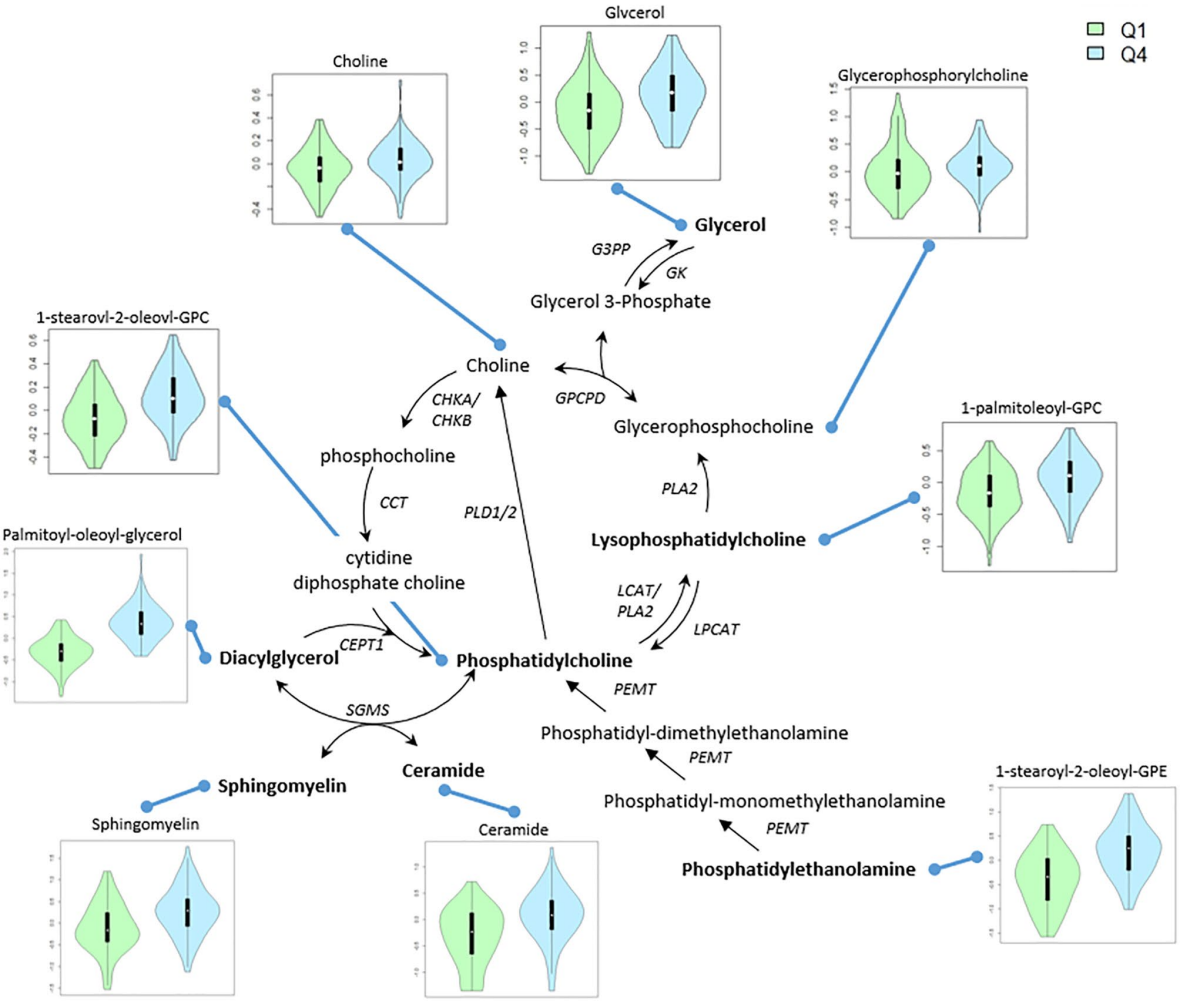
Measured Metabolites along Phosphatidylcholine Biosynthesis (CDP-choline pathway) and Catabolism Pathways. Shown are the distributions of measured metabolites in these pathways in individuals in quartiles 1 and 4 of TG. Where multiple metabolites could be described under the same heading, the most statistically significant is displayed. A bolded metabolite label indicates statistical significance based on the main linear model. *CCT: CTP:choline-phosphate cytidylyltransferase; CEPT1: choline/ethanolaminephosphotransferase1; CHKA: choline kinase alpha; CHKB: choline kinase beta; G3PP: glycerol-3-phosphate phosphatase; GK: glycerol kinase; GPCPD: glycerophosphocholine diesterase; LCAT: phosphatidylcholine-sterol acyltransferase; LPCAT: lysophospholipid acyltransferase; PLA2: phospholipase A2; PEMT: phosphatidylethanolamine N-methyltransferase; PLD1: phospholipase D; SGMS1: sphingomyelin synthase.*

There were 99 lipid metabolites associated with TG at an FDR q < 0.01 in at least one of the three models (main model [unadjusted for adiposity], adjusted for BMI, or adjusted for waist-to-hip ratio [WHR]; Supplementary Table S2), and 93 of these were statistically significant in our main model. The strongest associations were with diacylglycerols, including palmitoyl-oleoyl-glycerol (β 0.67, q = 5.4E−40) and oleoyl-oleoyl-glycerol (β 0.66, q = 1.4E−38), which is consistent with expectation as they are byproducts of TG hydrolysis and precursors of TG synthesis. Among the significantly associated metabolites, the subpathway most represented was diacylglycerols (n = 28 metabolites for TG analysis; Fig. 1). Metabolites in this subpathway, as well as monoacylglycerols (n = 10 metabolites) were positively associated with TG. There were 34 significant metabolites among glycerophospholipids, predominantly phosphatidylethanolamines, lysophospholipids, and phosphatidylcholines (n = 11, 10, and 7 metabolites, respectively). The relationship between some of these metabolites is shown in Fig. 2, which depicts the pathways connecting glycerol, the backbone of the triglyceride molecule, to phosphatidylcholine and related molecules. Throughout this pathway, higher median values of the measured metabolites can be seen in those in the highest compared to the lowest quartile of TG in our data, with 7 of the 9 metabolites depicted significantly associated with TG.

**Fig. 3:**
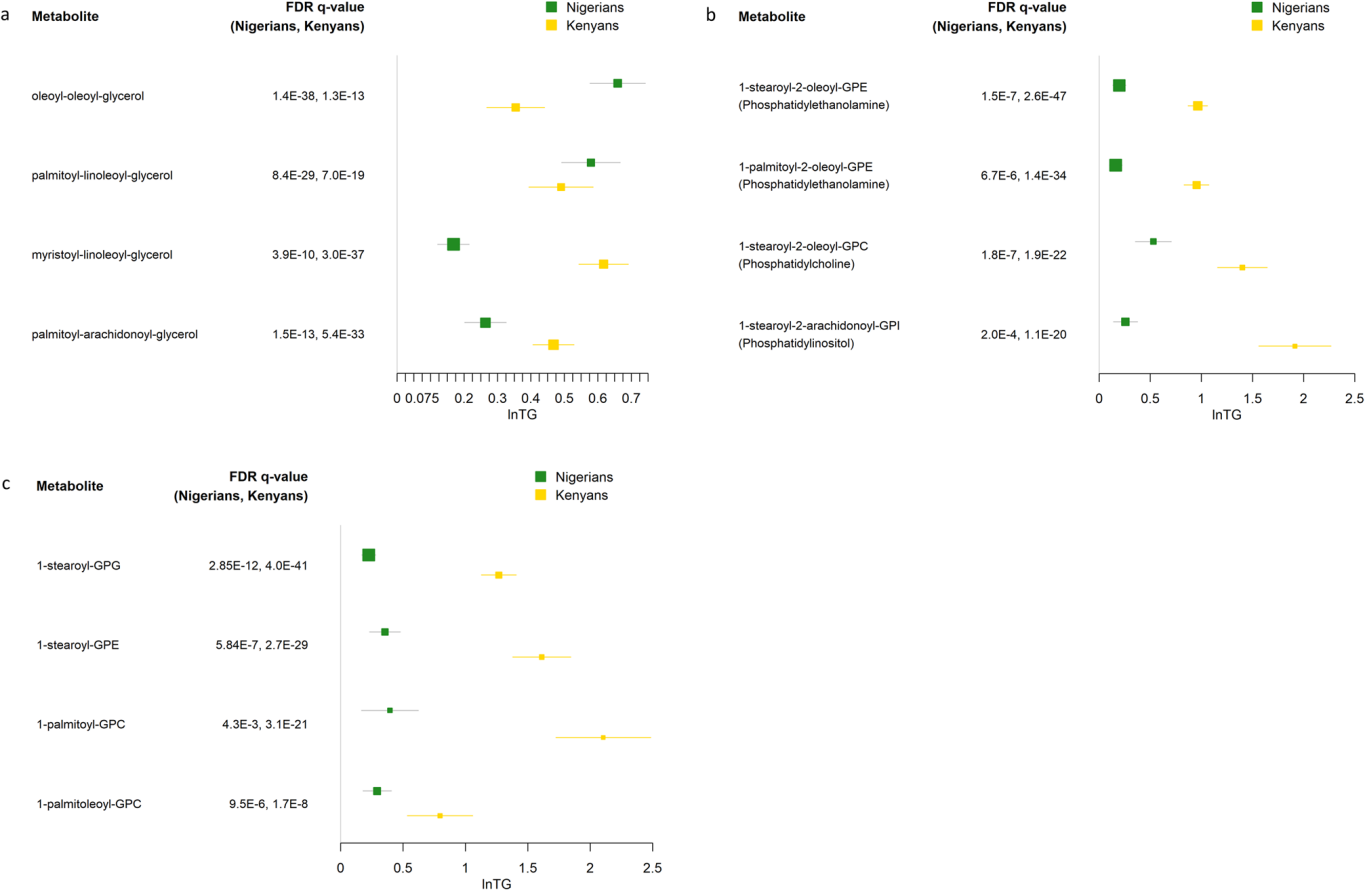
Association of Selected Metabolites with TG in Nigerians and Kenyans. Forest plots show the association of selected phospholipids and TG in Nigerians (discovery) and Kenyans (replication). Full replication results available (Supplementary Table 3). Shown are select associations for Diacylglycerols (a), Phosphotidylcholine, Phosphatidylethanolamine, and Phosphatidylinositol (b), and Lysophospholipids (c) with natural log-transformed TG.

Of the 99 lipid metabolites that were associated with TG, 80 were detected in our replication sample of 199 Kenyans (Supplementary Table S1). 73 of the metabolite-TG associations replicated (same direction of association and q < 0.05; Supplementary Table S3). 65 replicated at a more stringent level of statistical significance (Bonferroni corrected q of 0.05/80 = 0.000625). While each of the diacylglycerols evaluated replicated (q range: 3.4E−6 to 3.0E-37), the patterns of association across diacylglycerols varied, with substantial differences in effect sizes (Fig. 3a). For instance, among Nigerians, the most statistically significantly associated diacylglycerol was oleoyl-oleoyl-glycerol (β = 0.66; q = 1.4E−38) with a weaker association among Kenyans (β = 0.35; q = 1.3E−13). The most statistically significant association among Kenyans was for myristoyl-linoleoyl-glycerol (β = 0.62; q = 3.0E−37), with an effect size that was three times that among Nigerians (β = 0.17; q = 3.9E-10). Associations across phosphopholipids including phosphatidylcholine, phosphatidylethanolamine, and phosphatidylinositol all replicated (Fig. 3b), with stronger effect sizes and greater statistical significance among Kenyans compared to Nigerians. A similar pattern was observed among lysophospholipids, where the most statistically significant association for both Nigerians and Kenyans was for 1-stearol-GPG (q = 2.9E−12 [Nigerians], q = 4.0E−41 [Kenyans], Fig. 3c). Notably, the average of the absolute value of the betas for the tested TG-metabolite associations were larger among Kenyans (0.798 [Kenyans] vs. 0.246 [Nigerians], as were the standard errors (0.101 [Kenyans] vs. 0.046 [Nigerians]), potentially as a consequence of the markedly higher TG concentration in our Kenyan participants (Supplementary Table S1). We further evaluated this heterogeneity by meta-analyzing the Nigerian and Kenyan data: for 59 of the 80 metabolites tested there was evidence for heterogeneity (*p* < 0.05 for Cochran’s Q test; Supplementary Table S3). A comparison of median values of metabolites by TG quartile along the pathways depicted in Fig. 2 was also conducted in East Africans (Supplementary Fig. 1). Of the 8 metabolites measured along these pathways, 6 were statistically significantly associated with TG. In contrast to the West Africans, glycerol was not associated with TG concentration, but glycerophosphocholines were.

### Overrepresentation analysis and annotation of significantly associated lipid metabolites

To further evaluate the significantly associated metabolites, we performed an overrepresentation analysis using *MetaboAnalyst 5.0*. Of the 99 TG-associated metabolites, 65 could be matched to PubChem IDs and were included in the overrepresentation analysis. Diacylglycerols (q = 3.3E−18) and monoacylglycerols (q = 3.5E−12) were significantly enriched among the TG-associated metabolites (Table 1). Other overrepresented metabolite subclasses included diacylglycerophosphoethanolamines, monoacylglycerophosphocholines, ceramide phosphocholines, and diacylglycerophosphocholines. In addition to subclasses, metabolites can be characterized according to “main” and “super” chemical classifications. Results using these classifications provide a similar, but less detailed result (Supplementary Tables 4–5).

**Table 1:** Overrepresented metabolite subclasses among TG-associated metabolites.

	Total	Expected	Hits	Raw P	FDR
Diacylglycerols	531	0.18	13	5.7E−21	4.1E−18
Monoacylglycerols	15	0.00	5	1.1E−14	3.8E−12
Diacylglycerophosphoethanolamines	972	0.32	8	1.5E−09	3.5E−07
Monoacylglycerophosphocholines	46	0.02	3	5.3E−07	9.7E−05
Ceramide Phosphocholines	8	0.00	2	3.1E−06	4.5E−04
Diacylglycerophosphocholines	1300	0.43	5	7.6E−05	9.2E−03

Ingenuity Pathway Analysis (IPA) of TG-associated metabolites identified upstream regulators (Table 2; shown *p* < 1E–6). Five upstream regulators were predicted to play a role in the observed associations, and 3 of these were enzymes (ALDH1L1, SIRT6, and MGLL). ALDH1L1 and MGLL mainly target fatty acid esters and fatty acids while SIRT6 targets lysophosphotidylcholine in addition to fatty acid esters. C1QTNF2, a protein involved in the regulation of lipid metabolism in adipose tissue and liver, targets another class of phospholipids: phosphatidylethanolamines.

**Table 2:** Predicted Upstream Regulators (*p* < 1E-6) of TG-associated Metabolites (IPA©).

Upstream regulator (Molecule Type)	*P*-value of Overlap*	Target molecules in dataset	Associated disease(s)
ALDH1L1 (*enzyme*)	9.07E-11	1–22:6(4Z,7Z,10Z,13Z,16Z,19Z) monoacylglycerol 1-oleoylglycerol 1-palmitoylglycerol 2-linoleoylglycerol 2-oleoylglycerol	Alzheimer’s disease
SIRT6 (*enzyme*)	3.91E-09	1–16:0 lysophosphatidylcholine 1–16:1(9Z) lysophosphatidylcholine 1–18:0 lysophosphatidylcholine 1–22:6(4Z,7Z,10Z,13Z,16Z,19Z) monoacylglycerol 2-linoleoylglycerol,glycerol	hypercholesterolemia, diabetes mellitus, heart failure, hyperglycemia, chronic inflammation (among others)
Triacsin C (*chemical reagent*)	8.02E-09	1–16:0 lysophosphatidylcholine 1-stearoyl-2-oleoylphosphatidylcholine 16:0/20:4(5Z,8Z,11Z,14Z) phosphatidylethanolamine d18:0/18:0 dihydrosphingomyelin Palmitoylsphingomyelin	–
C1QTNF2 (*other*)	1.51E-08	16:0,22:6 phosphatidylethanolamine 18:0/18:2(9Z,12Z) phosphatidylethanolamine dipalmitoyl phosphatidylethanolamine Glycerol	Obesity
MGLL (*enzyme*)	1.01E-07	2-linoleoylglycerol 2-oleoylglycerol Arachidonic acid Glycerol	Weight gain, cardiomyopathy, liver cirrhosis, insulin resistance (among others)

*P value for a Fisher’s exact test of an overlap between TG-associated metabolites and metabolites regulated by an upstream regulator.

### Sensitivity analyses

Given the role that adiposity could play in either confounding or mediating associations between TG and lipid metabolites, we considered models that adjusted for either BMI (a measure of overall adiposity) or WHR (a measure of central obesity) or were unadjusted for adiposity. Correlation between the effect estimates of the adiposity unadjusted and adjusted models was very high (unadjusted vs. BMI-adjusted: 0.995; unadjusted vs. WHR-adjusted: 0.996). The minor differences between TG-metabolite associations across models gave little to no evidence for mediation or confounding of associations by adiposity. To assess whether these metabolites may be more strongly associated with other serum lipids, we also evaluated the association of each of our lead TG-associated metabolites with HDL-c and LDL-c. Of the 99 TG-associated metabolites, 11 were more strongly associated with LDL-c and 1 was more strongly associated with HDL-c than they were with TG (Supplementary Table 6).

**Fig. 4:**
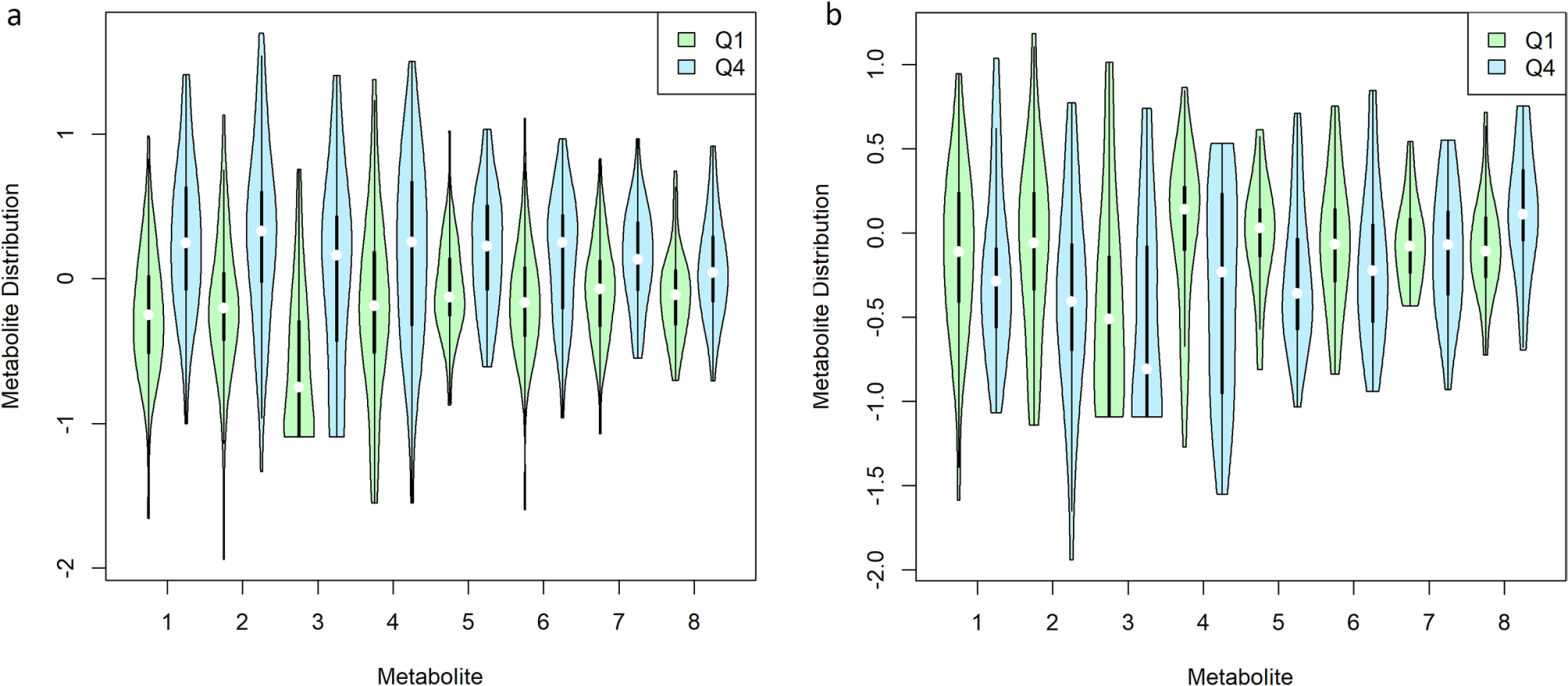
Distribution of Fatty Acyl Carnitines by TG Quartile by T2D Status. Distribution of age- and sex-adjusted values of selected fatty acyl carnitines in the lowest and highest quartiles of TG in T2D Controls (**a**) and Cases (**b**). Metabolites included were associated with TG at FDR p < 0.01 among T2D Controls.

Given that T2D is associated with perturbation of TG levels (high TG levels are common in T2D), we also evaluated the associations between TG and metabolites separately in individuals with and without T2D. Interestingly, of the 11 fatty acyl carnitines tested, 8 were associated at q < 0.01 among T2D controls (Fig. 4a), as was carnitine; none of these were associated among cases (Fig. 4b). In the overrepresentation analysis using subclassifications in *MetaboAnalyst*, both T2D cases and controls showed overrepresentation of metabolites associated with TG metabolism (diacylglycerols and monoacylglycerols) and phospholipids (diacylglycerophosphoethanolamines), but fatty acyl carnitines were overrepresented only among the T2D controls (Supplementary Table 7; note, not all metabolites included in association analyses could be matched to annotations for use in *MetaboAnalyst*). Similar findings were observed using the main classifications, with an overrepresentation of fatty esters among controls that was not observed in cases (4 hits in controls vs. no hits among cases). Importantly, the association between these metabolites and TG were dramatically different in the two groups. For instance, dihomo-linolenoylcarnitine was strongly associated with TG in controls (β 0.28, q = 1.8E−6), while there was no association among cases (β-0.14, q = 0.32). A strong association with carnitine among controls (β 0.45, q = 1.0E−4) was not observed in cases (β-0.01, q = 0.94).

### Association between the most significant TG-associated lipid metabolites and cardiometabolic traits

**Fig. 5:**
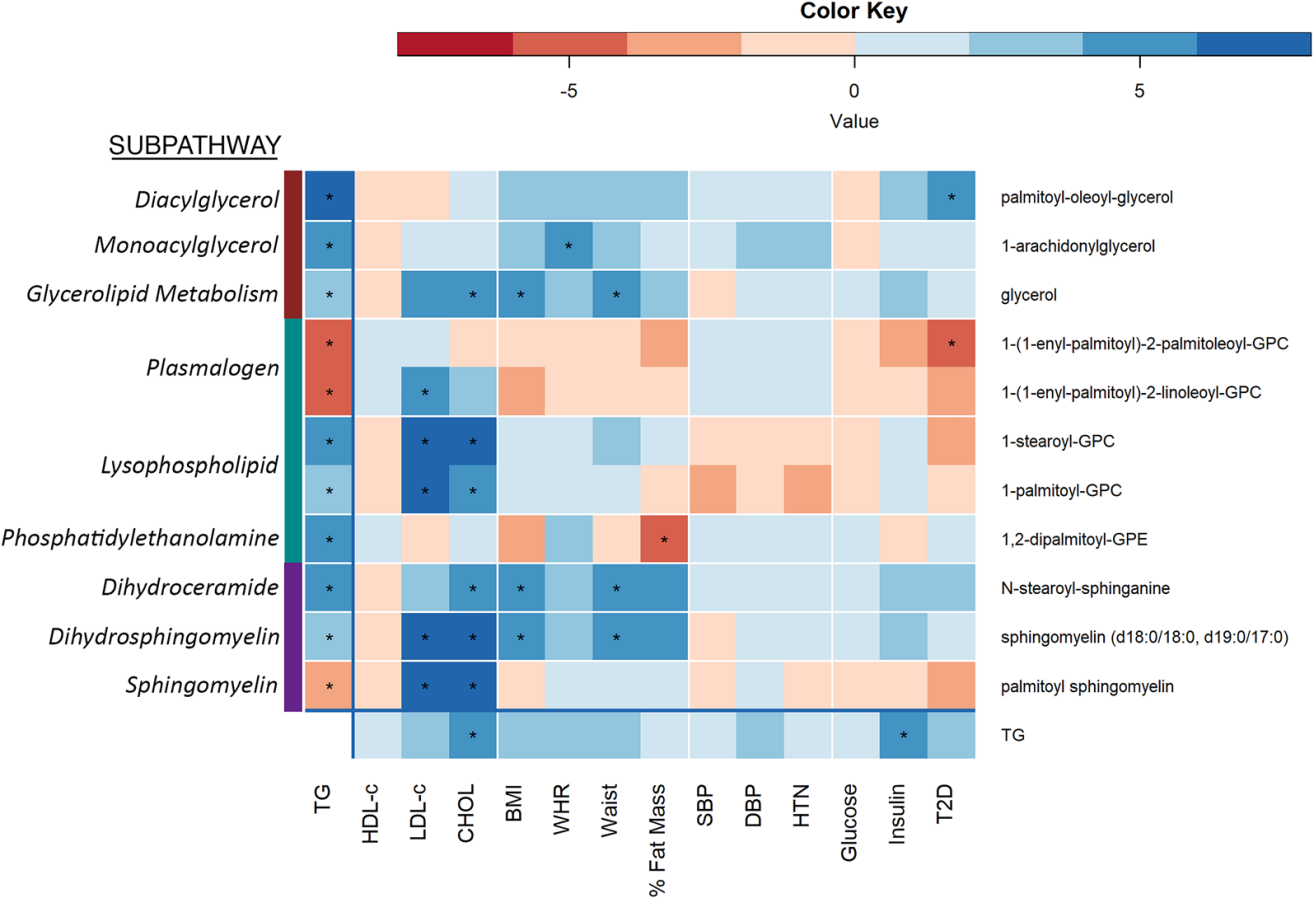
Association of TG-associated Metabolites across Cardiometabolic Traits. Shown is a heat map of the T-values of the associations between selected TG-associated metabolites and cardiometabolic traits. Asterisks indicate statistical significance. The color bar along the Y axis represents the subpathway of metabolite. Metabolites selected included the most statistically significant positive and inverse association for each trait (if it met the threshold for statistical significance) as well as any with a T-value < -5 or > 5. For full association results, see Supplementary Table 8.

Since TG concentration is a strong predictor of cardiometabolic disease risk, we evaluated the association of our lead metabolites with 13 cardiometabolic traits (Fig. 5; Supplementary Table 8). As expected, the strongest associations were with other lipids traits (HDL-c, LDL-c, and total cholesterol concentration [CHOL]), followed by traits related to adiposity (BMI, waist circumference, WHR, and percent body fat). Notably, there were no statistically significant associations of these TG-associated metabolites with blood pressure traits (systolic blood pressure [SBP], diastolic blood pressure [DBP], or hypertension [HTN]), fasting glucose, or fasting insulin. Sphingomyelin (d18:0/18:0, d19:0/17:0) in the dihydrosphingomyelin pathway was associated with the largest number of traits, with positive associations with LDL-c, CHOL, BMI, and waist circumference. We also observed that different compounds within a subpathway displayed unique patterns of association. For example, while the diacylglycerols were both strongly and inversely associated with HDL-c, only one was associated with T2D. Similar differences in HDL-c and T2D associations were observed with the plasmalogens as well. Importantly, there were metabolites that were more strongly associated with each of the lipids, obesity, and T2D traits than TG was, suggesting that these are more proximal to the cardiometabolic trait than TG. The only trait for which TG was more strongly associated than the TG-associated metabolites was insulin. While there was some overlap between metabolites associated with serum lipids and adiposity, there were metabolites that only were significantly associated with one or the other, offering the potential to disentangle the broad associations of TG with cardiometabolic traits.

### Mediation analysis

**Fig. 6:**
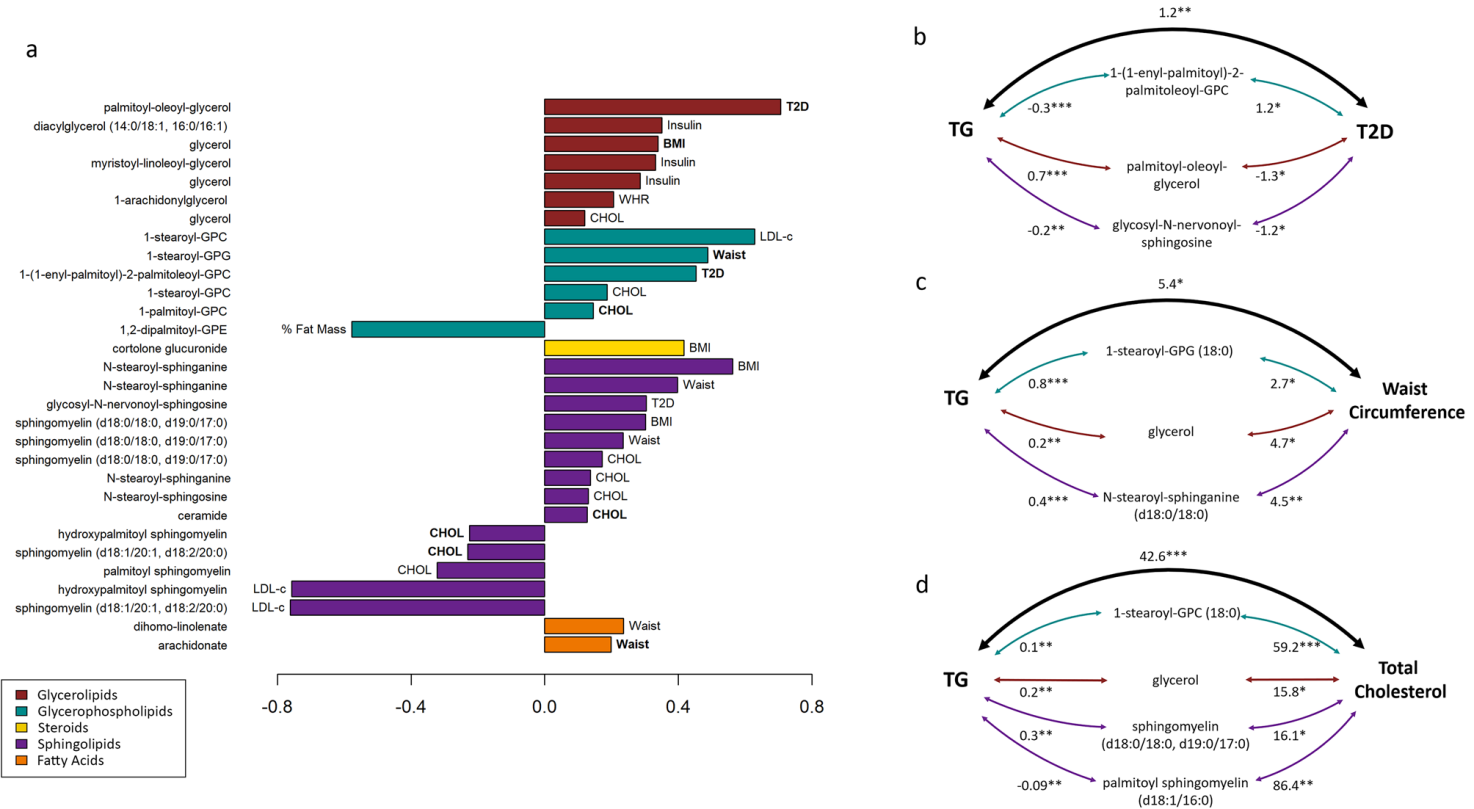
Mediation of the association between TG and Cardiometabolic Traits by TG-associated metabolites. a. Proportion of the TG-cardiometabolic trait association mediated by the listed metabolite. Associations that were totally mediated by the metabolite (i.e., a direct effect of TG and the cardiometabolic trait was no longer statistically significant after accounting for the indirect effect of the metabolite) are indicated with bolding of the trait label. For full results, see Supplementary Table 9. b-d. The figures represent the associations between TG and T2D (b), waist circumference (c), and total cholesterol (d) along with significant, independent mediators of those associations. The β coefficient for a given association is annotated on the arrows, with p-values represented by asterisks: *p < 0.05, **p < 10^–3^, ***p < 10^–6^. The color of the arrow indicates the subpathway of the metabolite. Results for all tested traits available in Supplementary Table 10.

We next evaluated whether the lead metabolites that were associated with cardiometabolic traits mediated the associations between TG and those cardiometabolic traits. At least partial mediation of the TG-cardiometabolic trait by the relevant metabolite was observed for all 30 TG/metabolite/trait combinations that were tested (see Methods for selection criteria; Fig. 6; Supplementary Table 9). Total mediation of the TG-trait association was observed in 9 associations: after accounting for the indirect effect of the metabolite on the TG-trait association, no significant direct association between TG and the trait remained. Analyses to assess the sensitivity of mediation results to potential confounding through evaluating correlation in the residuals between the mediator and the outcome (see Methods for further details) indicate that the most robust of our results were for sphingomyelin, hydroxypalmitoyl sphingomyelin, and palmitoyl sphingomyelin with LDL-c and CHOL, as well as 1-stearoyl-GPC with LDL-c (Supplementary Table 10). For each of these, the correlation in the residuals (rho) between the mediator and the outcome (i.e. representing an unadjusted confounder) would have to be at least 0.4 for the mediation effect to be reduced to 0. We further investigated traits for which multiple metabolites were found to mediate the association with TG to determine whether the association between these metabolites and the outcome were independent of each other or whether the association with some metabolites was mediated by another of the metabolites. The association between TG and T2D was mediated independently by 3 metabolites, which each represented separate subpathways: glycerolipids, phospholipids, and sphingolipids (Fig. 6b. After adjusting for these metabolites, the association between TG and T2D was no longer statistically significant. Similarly, the TG-waist circumference association was mediated by 3 metabolites from the same 3 subpathways (Fig. 6c), and the association was completely mediated by these metabolites. Four metabolites from the same 3 subpathways partially mediated the association between TG and CHOL (Fig. 6d), with the TG association remaining highly significant even after adjustment for these metabolites (p = 4.4E−10). The associations with BMI and LDL-c were mediated completely by 3 metabolites, though the subpathways differed: glycerolipids (BMI), sphingolipids (BMI, LDL-c), glycerophospholipids (LDL-c), and steroids (BMI). Insulin, WHR, and % fat mass were each partially mediated by only a single metabolite. Association results of the independent, mediating metabolites for each trait are given in Supplementary Table 10.

## Discussion

We utilized a lipidomic approach towards identifying the lipid metabolites that are co-dysregulated with TG, a known risk factor for atherosclerosis and multiple cardiometabolic disorders. Our major findings included identifying a set of 99 TG-associated metabolites (notably diacylglyerols, monoacylglycerols, phosphatidylethanolamines, and lysophospholipids) in our sample of Nigerians, most of which replicated in an independent cohort of Kenyans. Higher TG was associated with higher metabolite levels for all metabolites except for plasmalogens, which were inversely associated with TG. We also found differences in TG-metabolite associations by T2D status, with T2D controls showing associations with fatty acid carnitines that were not observed in T2D cases. The metabolites with the strongest TG-associations also were significantly associated with other commonly measured lipid traits (CHOL, HDL-c, and LDL-c) and adiposity traits, but not with blood pressure traits (SBP, DBP, or HTN), fasting glucose, or fasting insulin.

The strongest associations with TG in our data were for diacylglycerols and monoacylglycerols. This result is anticipated, given that these compounds are TG hydrolysis products and TG synthesis intermediates. Notably, however, effects of these compounds may not be limited to their relationship with TG. For example, monoacylglycerol 1-linoleoylglycerol (18:2), which was positively associated with TG in our data, was shown to have an association with incident coronary heart disease that was independent of TG^16^. We also saw higher concentrations of glycerol, the backbone of tri-, di-, and monoacylclygerols, with higher TG concentration. Higher concentrations were also observed in the measured metabolites in the pathway connecting glycerol to phosphatidylcholine, showing a consistent effect on a network of interconnected reactions with higher TG concentration.

Glycerophosphocholines (GPC) were prominent among the associated metabolites, with positive associations observed with TG. In our overrepresentation analysis, we identified significant overrepresentation of both monoacylglycerophosphocholines and diacylglycerophosphocholines among the TG-associated metabolites. Circulating GPC has been associated previously with TG (with varying directions depending on the particular GPC), with some evidence of a GPC mediating the relation between visceral fat and TG^17^. GPC can be metabolized by the gut flora to trimethylamine N-oxide (TMAO), which has been associated through various lines of evidence with increased cardiovascular disease and metabolic disorders (reviewed in^18^ and ^19^, respectively). Given some encouraging findings related to neurovascular health and cognitive decline^20^, α-GPC has been used as a supplement to provide precursors of choline, an essential nutrient, to enhance brain cholinergic function. α-GPC supplementation has recently been associated with increased stroke risk^21^. In animal models, α-GPC supplementation leads to atherosclerosis, with evidence of increased plasma TMAO concentration^22^. In our study population, no individuals were supplementing with α-GPC. Key dietary sources of choline include eggs, beef, and fish, but the effects of increased dietary choline on TMAO levels in humans are unclear^18^, potentially because the production of TMAO is dependent on the gut microbiome, the composition of which is determined in a complex way by the diet.

Plasmalogens were the only metabolites that were significantly inversely associated with TG in the present study. Plasmalogens are a class of glycerophospholipids commonly found in cell membranes and have a unique structure compared to other phospholipids. They are thought to facilitate a role in protection from oxidative stress and in the structure of the cell membrane^23^. Decreased plasmalogen concentration has been associated with cardiovascular disease and other conditions^23,24^. Therefore, the inverse relationship we observed in this study is consistent with elevated TG and/or decreased plasmalogen concentrations conveying increased cardiovascular risk.

We found an overrepresentation of associations of fatty acyl carnitines with TG among participants without T2D. Serum acyl carnitines have previously been associated with TG^25,26^. Serum acyl carnitines have also been previously associated with both cardiovascular disease^25,27^ and with T2D and related traits^28–30^. It has even been suggested that accumulation of stearoylcarnitine in β cells impairs energy metabolism, oxidative phosphorylation, the TCA cycle, and insulin synthesis^31^. We note that stearoylcarnitine was positively associated with TG in our T2D controls, though the FDR value was just over our threshold for statistical significance [FDR 0.03]. It is thought that the accumulation of acyl carnitines occurs as a result of inefficient long-chain fatty acid β oxidation^29^. The positive association that we observed between fatty acyl carnitines and TG may have been obscured among T2D cases due to metabolic dysregulation accompanying disease processes. Interestingly, in addition to choline as described above, carnitine is a key dietary precursor in the formation of TMAO, and red meat, fish, eggs, and dairy are rich sources for both nutrients^32^.

We identified 5 predicted upstream regulators of our associated metabolites: ALDH1L1, SIRT6, triacsin C, C1QTNF2, and MGLL. Of particular interest, some of these upstream regulators have been associated with cardiometabolic traits (though not TG), such as hypercholesterolemia, T2D, heart failure, and hyperglycemia (SIRT6); obesity (C1QTNF2); and weight gain, cardiomyopathy, liver cirrhosis, and insulin resistance (MGLL). The gene encoding *SIRT6* is only 1 kb from well-established TG locus *CREB3L3*^33,34^. This gene encodes CREBH, a transcription factor that regulates genes encoding factors in lipid and glucose metabolism^34^, and variants in *CREB3L3* have been significantly associated in multiple GWAS of TG^35–37^. Monoacylglycerol esterase (*MGLL*) is a critical enzyme to fatty acid metabolism and catalyzes the conversion of monoacylglycerol to free fatty acids and glycerol. *Mgll* knockout mice have elevated monoacylglycerols in their tissues and do not experience elevated plasma or liver TG or insulin resistance when fed a high fat diet^38^.

Measured serum TG represents the influence of many different pathways. Thus, the observed broad associations of TG may represent the influence of different pathways for different outcomes. Analysis of the associations of our lead metabolites with cardiometabolic traits in our data supports this hypothesis. For instance, most, but not all, of the metabolites in Fig. 5 were significantly associated with other serum lipids, but not with the same pattern. Most metabolites were associated with either LDL-c and CHOL (strongest with lysophospholipids and sphingomyelins) or with HDL-c (strongest with plasmalogens and diacylglycerols). Metabolites in the diacylglycerol and plasmalogen pathways were the only ones to be significantly associated with T2D. Although glycerol and metabolites in the dihydroceramide and dihydrosphingomyelin subpathways were associated with both measures of adiposity and serum lipids, a phosphatidylethanolamine and a monoacylglycerol were only associated with adiposity measures and not serum lipids. Subsequent analysis into the number of independent metabolites mediating the association of TG with cardiometabolic traits reveals that the association between TG and some of these traits (T2D, BMI, Waist Circumference, LDL-c, and CHOL were mediated by metabolites from multiple subpathways, particularly glycerolipids, sphingolipids, and glycerophospholipids. Although these sub-pathways are highly interconnected, these findings emphasize that TG concentration summarizes flow from multiple pathways, which may contribute in differing ways to separate cardiometabolic traits. Disentangling TG associations at this level may be useful for better understanding of the underlying biology of cardiometabolic traits, and future work should further explore the role of some of these key metabolites with these traits.

Interestingly, neither TG nor any of the associated metabolites was significantly associated with blood pressure traits. In a study of European ancestry individuals (n = 2786), 8 phospholipids (with 4 included among our lead metabolites) were positively associated with TG and blood pressure traits^11^, in contrast to our findings of no association between these metabolites and blood pressure metabolites. This study also reported stronger associations between these phospholipids and T2D-related traits than we found in our study. These differences may reflect the larger sample size in that study, but ancestry-related differences in the associations between TG and cardiometabolic traits have been previously reported ^15^. An analysis of the associations with TG and cardiometabolic traits across ancestry groups reported differing associations in West African vs. European ancestry populations between TG and a variety of traits. Associations between TG and BP traits were weaker in West African vs European ancestry individuals in a variety of different environmental contexts: a positive, significant association with HTN among European ancestry individuals was weaker and not significant among West African individuals and the regression coefficients for a positive association with SBP among those with West African ancestry was less than half of what was reported for European ancestry individuals. Weaker associations were also observed in West African vs. European ancestry individuals with adiposity traits^15^.

Sphingomyelin (d18:0/18:0, d19:0/17:0) was significantly associated with LDL-c, CHOL, BMI, and waist circumference in our data. The associations with LDL-c and CHOL, in particular, are expected, as 63–75% of plasma sphingomyelin is in LDL-c and VLDL-c particles, and it is the second most abundant polar lipid in plasma lipoproteins^39^. Sphingomyelin is synthesized from phosphatidylcholine and ceramide, both of which were significantly associated with TG in our data, showing consistently higher concentration throughout the sphingomyelin synthesis pathway with higher TG. Sphingomyelin had been considered mainly a structural lipid, related to membrane fluidity, and as a “depot fat”, but its role in metabolic signaling is being more appreciated^40^. Sphingomyelin, dihydroceramides, ceramides, and lactosylceramides, all of which were positively associated with TG in our data, have been identified as part of a common signature of carotid plaques, with evidence for a role in inducing plaque inflammation^41^.

This study has several strengths, including a study design that included discovery and replication samples, use of a state-of-the-art metabolomics assay platform, participants from underrepresented populations, and analysis of other cardiometabolic risk factors and outcomes. Another strength is the mediation analysis which facilitated our understanding of how observed TG-cardiometabolic traits associations are mediated by lipid metabolites. We also note the limitations, which include the fact that metabolites provide a snapshot of products of pathways influenced by multiple sources. As such, incorporating data from one of those other sources, for example dietary intake or the gut microbiome, would have provided a fuller picture of the relationships described here. The study focused only on lipid metabolites and it is possible that non-lipid metabolites and metabolites not detected in our study may also be associated with TG. The study samples were drawn from an ongoing study genetic epidemiology study of T2D, and, thus, participants are not necessarily representative of the general population at the study sites or other African populations.

This lipidomics study of sub-Saharan African adults identified significant TG-lipid metabolite associations involving a variety of lipid subpathways. In addition to expected associations in lipid metabolites related to triglyceride synthesis and hydrolysis, we identified associations with other lipids classes, including phosphatidylethanolamines, phosphatidylcholines, lysophospholipids, and plasmalogens. T2D disease processes may obscure associations with TG that can be found when looking only at those without T2D, as with fatty acyl carnitines in our data. The pattern of associations of metabolites with a variety of cardiometabolic outcomes suggest that different pathways may underlie the associations between TG and these traits. Our findings yielded important insights into lipid metabolites that covary with TG in an African population and add to the growing literature on metabolomics and cardiometabolic disorders.

## Methods

Study participants were drawn from the AADM study, which has been previously described^42–44^. Briefly, AADM is a genetic epidemiology study of T2D and related disorders enrolling participants from university medical centers in Nigeria, Ghana, and Kenya. For the work described here, 308 participants with and without T2D were enrolled from Ibadan, Nigeria. All participants completed a clinical examination that included standard clinical measurements and blood sampling. Serum TG was determined enzymatically on fasting samples either with the COBAS Integra 400 Plus or Modular-E analyzers (Roche Diagnostics, Indianapolis, IN). Methods were standardized to appropriate reference methods (isotope dilution mass spectrometry). None of the participants were using lipid-lowering medication. T2D status was determined based on a fasting plasma glucose ≥ 126 mg/dl, self-report of physician’s diagnosis of T2D, or use of glucose-lowering medication.

For each metabolite, the raw values (i.e., unnormalized peak areas) quantified in the experimental samples were divided by the median of those samples in each instrument batch, giving each batch and thus the metabolite a median of one. For each metabolite, the minimum value across all batches in the median scaled data was imputed for the missing values. The batch-normalized and imputed data was transformed using the natural log. Out of 1116 metabolites detected, 480 that were annotated as part of the Lipids Super Pathway were retained for analysis.

Analyses used natural log-transformed TG as a quantitative trait. Linear regression was conducted in R 3.4.4 (https://www.R-project.org/) using *lm* with adjustment for sex and age. As adiposity could mediate or confound the association between TG and lipid metabolites, models were fitted with and without adjustment for general adiposity (BMI) and central adiposity (WHR). To determine the association of our lead metabolites with other clinically important serum lipids, we evaluated linear regression models of each of these metabolites on log-transformed HDL-c and LDL-c using the same covariate adjustments as above. Relative strength of association was described based on comparison of P-values between models (prior to FDR adjustment). To adjust for the potential effect of T2D on the associations between metabolites and TG, T2D status was also included as a covariate in all models. Additionally, regression models were run in data stratified by T2D status to check for a different association by disease state. Associations with FDR q < 0.01 was considered statistically significant.

Metabolites that were associated with TG were further evaluated to detect patterns and functional annotation of interest. Overrepresentation analysis was conducted in MetaboAnalyst 5.0^45^ (https://www.metaboanalyst.ca/), which uses a hypergeometric test to evaluate whether metabolites in a super-, main-, or subclassification are observed more than would be expected by chance within a given list of metabolites. We included all metabolites associated with TG at FDR q < 0.01 in this analysis. TG-associated metabolites were further evaluated using Ingenuity Pathway Analysis^46^ (IPA, QIAGEN Inc, https://digitalinsights.qiagen.com/products-overview/discovery-insights-portfolio/analysis-and-visualization/qiagen-ipa/).

Statistically significant metabolite-TG associations were also tested in an independent sample of 199 participants enrolled from Eldoret, Kenya as part of the AADM study. Metabolite-TG associations were tested using the same methods as in the main analysis. A consistent direction of effect and a *p*-value < 0.05 was considered evidence for replication. We also report replication by a more stringent threshold, with Bonferroni correction for multiple tests (0.05/number of metabolites tested). Heterogeneity in discovery and replication samples was assessed through meta-analysis of TG-associated metabolites (using the R package *meta*), with p < 0.05 for Cochran’s Q test considered evidence for statistically significant heterogeneity across samples.

To test for associations of the most significant TG-associated metabolites with cardiometabolic traits, we evaluated linear and logistic mixed models for: HDL-c, LDL-c, CHOL, BMI, WHR, waist circumference, percent fat mass, SBP, DBP, HTN, fasting plasma glucose, fasting insulin, and T2D. All models were adjusted for age and sex. The 93 metabolites that were significantly associated with TG in our main analysis were evaluated. Statistical significance was set at *p* < 4.14E−5 (*p* < 0.05/ [13 traits × 93 metabolites]). To test whether these metabolites mediated the associations between TG and TG-associated cardiometabolic traits, we conducted analyses of 30 TG/metabolite/cardiometabolic trait combinations meeting the following criteria: the TG-cardiometabolic trait association was *p* < 0.05 and the metabolite-cardiometabolic trait was statistically significant (as described above). These analyses were conducted using the R package *mediation* version 4.5.0 (https://cran.r-project.org/web/packages/mediation/index.html). Total (vs. partial) mediation of the TG-cardiometabolic association was declared if the p-value for the indirect effect of TG was *p* < 0.05 and the p-value for the direct effect of TG was *p* > 0.05. Metabolites that were shown to significantly mediate a cardiometabolic trait were further evaluated using the same strategy to determine if metabolite-trait associations were mediated by another associated metabolite and to evaluate independence of the metabolites.

Sensitivity of the mediation analyses to unmeasured confounding was evaluated using medsens() within the R *mediation* package version 4.5.0, as previously described^47^. This analysis assesses the potential impact of different values of unmeasured confounding, described as the correlation between the residuals in the model of the mediator and of the outcome (rho). The key outcome of this sensitivity analysis is the value of rho at which the unmeasured confounding would result in an average mediation effect of 0. If this value (bounded by 1 and − 1) is close to 0, this can be interpreted to mean that a minor degree of unmeasured confounding would be sufficient to reduce the reported effect to 0, while larger numbers are better evidence for robustness of estimates. There are no specific thresholds to determine what qualifies as a result that is sensitive or robust to unmeasured confounding. Thus, we present the values of rho at which the observed association would be reduced to 0, with those small values to be likely sensitive to unmeasured confounding and those larger, such as 0.5, to be considered as relatively more robust.

Visualization of results was performed in R 4.2.1 using the packages *violplot* (https://cran.r-project.org/package=vioplot), *forestplot* (https://cran.r-project.org/package=forestplot), and *gplots* (https://cran.r-project.org/package=gplots; heatmap.2 function).

## Supplementary Information

Supplementary Figures.

Supplementary Tables.

## Data Availability

The datasets generated and analyzed during the current study will be made publicly available through the dbGaP with accession number phs001844.v1.p1.
