# Supplementary material for: Lipidomics profiling and circulating triglyceride concentrations in sub-Saharan African individuals
Source: Sci Rep. 2024 Sep 6;14:20834. doi: 10.1038/s41598-024-71734-3 (PMC11385232; doi:10.1038/s41598-024-71734-3)
Supplement: Supplementary file 1 — Supplementary Figures. [file 41598_2024_71734_MOESM1_ESM.docx]

*Supplemental FIgure*

**Lipidomics Profiling and Circulating Triglyceride Concentrations in African Individuals**

Amy R. Bentley; Ayo P. Doumatey; Jie Zhou; Lin Lei; Karlijn A.C. Meeks; Elisabeth F. Heuston; Charles N. Rotimi; and Adebowale A. Adeyemo

**Supplemental Figure 1: Measured Metabolites along Phosphatidylcholine Biosynthesis (CDP-choline pathway) and Catabolism Pathways among East Africans.** Shown are the distributions of measured metabolites in these pathways in individuals in quartiles 1 and 4 of TG among East African samples. Metabolites presented were selected to match those in the figure for West Africans (**Figure 2**) where possible: neither ceramide nor palmitoyl-oleoyl-glycerol were available in this dataset (palmitoyl-oleoyl-glycerol is replaced with oleoyl-oleoyl-glycerol in the above). A bolded metabolite label indicates statistical significance based on the main linear model. *Abbreviations: CCT: CTP:choline-phosphate cytidylyltransferase; CEPT1: choline/ethanolaminephosphotransferase1; CHKA: choline kinase alpha; CHKB: choline kinase beta; G3PP: glycerol-3-phosphate phosphatase; GK: glycerol kinase; GPCPD: glycerophosphocholine diesterase; LCAT: phosphatidylcholine-sterol acyltransferase; LPCAT: lysophospholipid acyltransferase; PLA2: phospholipase A2; PEMT: phosphatidylethanolamine N-methyltransferase; PLD1: phospholipase D; SGMS1: sphingomyelin synthase*


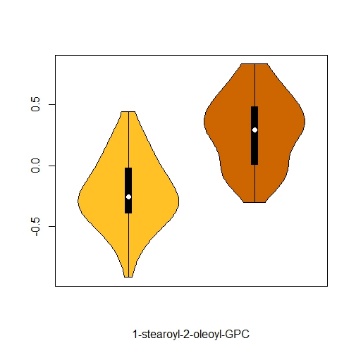

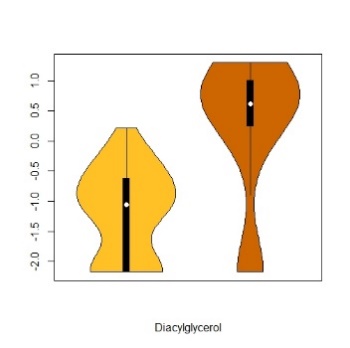

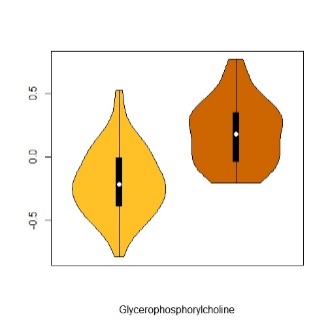

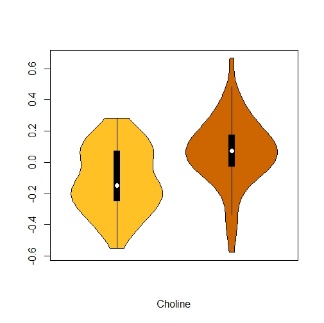

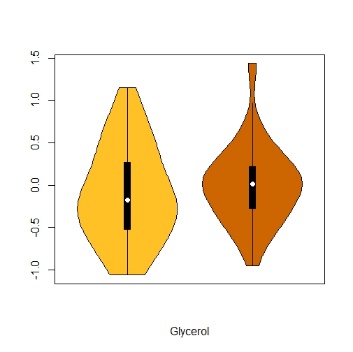

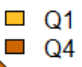

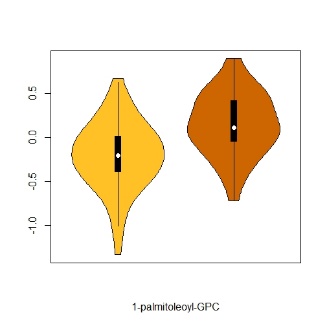

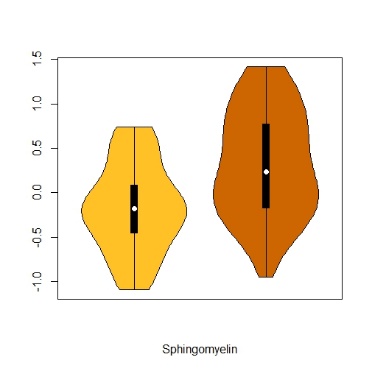

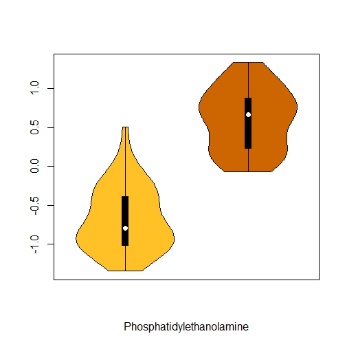


*CCT*

*CEPT1*

Choline

**Glycerophosphocholine**

**Lysophosphatidylcholine**

**Phosphatidylcholine**

phosphocholine

*GPCPD*

*CHKA/*

*CHKB*

*LPCAT*

*LCAT/*

*PLA2*

*PLA2*

Glycerol 3-Phosphate

Glycerol

*G3PP*

*GK*

Choline

1-palmitoleoyl-GPC

1-stearoyl-2-oleoyl-GPC

Glycerophosphorylcholine

*PLD1/2*

Glycerol

Ceramide

**Sphingomyelin**

*SGMS*

Oleoyl-oleoyl-glycerol

Sphingomyelin

cytidine

diphosphate choline

**Diacylglycerol**

**Phosphatidylethanolamine**

Phosphatidyl-dimethylethanolamine

Phosphatidyl-monomethylethanolamine

*PEMT*

1-stearoyl-2-oleoyl-GPE

*PEMT*

*PEMT*
